# Current status of nylon teeth myth in Tanzania: a cross sectional study

**DOI:** 10.1186/s12903-017-0462-6

**Published:** 2018-01-10

**Authors:** Emeria Abella Mugonzibwa, Febronia Kokulengya Kahabuka, Samwel Charles Mwalutambi, Emil Namakuka Kikwilu

**Affiliations:** 1 School of Dentistry, Department of Orthodontics Paedodontics and Community Dentistry, Muhimbili University of Health and Allied Sciences (MUHAS), P.O. Box 65014, Dar es Salaam, Tanzania; 2 Dental Department, Muhimbili National Hospital (MNH), P.O. Box 65000, Dar es Salaam, Tanzania

**Keywords:** Nylon teeth myth, Tooth bud gouging, Childhood diseases, Tanzania

## Abstract

**Background:**

Nylon teeth myth is a belief of associating infant illnesses with bulges on infants’ alveolus that mark the positions of underlying developing teeth and that it is necessary to treat the condition mainly by traditional healers to prevent infant death. The traditional treatment often leads to serious complications that may lead to infant death. Although the government instituted educational campaigns against the myth in 1980s to 1990s, to date, repeated unpublished reports from different parts of the country indicate continued existence of the myth. Therefore, this study aimed to assess the current status of the nylon teeth myth in Tanzania.

**Methods:**

The study population was obtained using the WHO Oral Health pathfinder methodology. A structured questionnaire inquired about socio-demographics as well as experiences with “nylon teeth” myth and its related practices**.** Odds ratios relating to knowledge and experience of the nylon teeth myth were estimated.

**Results:**

A total of 1359 respondents aged 17 to 80 years participated in the study. 614 (45%) have heard of nylon teeth myth, of whom 46.1% believed that nylon teeth is a reality, and 42.7% reported existence of the myth at the time of study. Being residents in regions where nylon teeth myth was known before 1990 (OR = 8.39 (6.50–10.83), *p* < 0.001) and/or hospital worker (OR = 2.97 (1.99–4.42), *p* < 0.001) were associated with having have heard of nylon teeth myth. Proportionately more residents in regions where nylon teeth myth was not known before 1990 (*p* < 0.001), the educated (*p* < 0.001) and hospital workers (*p* < 0.001) believed modern medicine, whereas, proportionately more residents in regions where nylon teeth was known before 1990 (*p* < 0.001), less educated (*p* < 0.001) and non-hospital workers (*p* < 0.001) believed traditional medicine to be the best treatment for symptoms related to nylon teeth myth respectively.

**Conclusion:**

The “nylon teeth” myth still exists in Tanzania; a substantial proportion strongly believe in the myth and consider traditional medicine the best treatment of the myth related conditions.

**Electronic supplementary material:**

The online version of this article (10.1186/s12903-017-0462-6) contains supplementary material, which is available to authorized users.

## Background

Nylon teeth myth is a belief of associating infant illnesses with bulges on infants’ alveolus that mark the positions of underlying developing teeth, especially canines. The commonest illnesses associated with the myth include repeated or long standing diarrhea, fevers, difficult sucking milk from mother’s breast, and itching in the mouth [1].The myth is named differently in different parts of East Africa, as summarized by Girgis and colleagues [2]. The believers assert that if no treatment is done, the infant is likely to die of the condition [3–5]. From early 1980s to date, the myth has been reported in Uganda [3, 4, 6–8] Tanzania [9–12], Sudan [13, 14], Ethiopia [15, 16] and Kenya [5, 17, 18], and Somalia [19] indicating signs of spreading from country to country, and from region to region.

The commonest form of traditional treatment as summarized in the review of studies by Johnston and Riordan [20] is gouging/removal of the underlying developing tooth germ. Rubbing of coarse herbs onto the gums has also been documented in Tanzania [12]. Gouging of the tooth germs is conducted by unprofessional people using crude instruments, and in most cases under unhygienic conditions. The immediate consequences of this form of treatment include – delayed or even denial of proper management of the underlying cause of the infant illness in question; excessive/uncontrolled bleeding leading to anaemia, introduction of infectious agents into the wounds that may lead to septicemia, meningitis, osteomyelitis and tetanus.These conditions are in most cases fatal [3, 4, 9]. The later consequences are mainly missing primary teeth corresponding to the tooth germs that were removed/gouged [1, 8], malformed teeth if the tooth germs were partially removed, and or hypoplasia of the permanent teeth if their tooth germs were traumatized during the process of gouging [15, 16, 19, 21]. Other complications include; midline shift to the affected side [16, 21]; distal eruption of permanent lateral incisors [15, 16]; failure of development of permanent canine and compound odontoma [6, 10, 16].

Due to these consequences, Tanzania initiated educational campaigns to discourage the myth and its associated practices through mass media and in health care facilities. To date, no published reports that show the existence or extinction of the myth, but repeated verbal reports about the myth continued to be heard in different dental professional meetings as well as among parents who bring their children for dental consultation at the Muhimbili Dental clinic. Therefore, this paper reports the current status of the nylon teeth myth in Tanzania.

## Methods

The study population was obtained using the WHO Oral Health pathfinder methodology [22]. Tanzania is divided into five geographical zones. Of the five zones, high prevalence of nylon teeth myth was previously reported in three zones. One region from each of these zones, was included in the study. In the other two zones where the belief had not been previously reported, a multicultural city and two regions furthest from the regions that were previously reported to have nylon teeth myth were conveniently selected. The strata of interest in the current study were hospital workers; teachers, traditional healers; adults of child bearing age (17–45 yrs); elders (46+ yrs). Four study sites from urban and 8 from rural areas were chosen. For each study site, 25 respondents from each stratum of interest were targeted. This gave a total of 1200 subjects. For each study site, the interviewers were led by the street or village leaders to school, health facility, known traditional healers’ homes and house to house for the rest of the study population until the desired number of subjects per stratum was attained or when all persons for a given strata had been interviewed.

A Kiswahili version questionnaire was used to inquire on demographic characteristics as well as experiences with nylon teeth myth and its related practices (Additional file 1). A field testing of the questionnaire was done to check for clarity and meaning of the questions. Muhimbili University of Health and Allied sciences Research and Ethical Committee granted the ethical clearance. Written informed consent was obtained from each participant.

In multivariate logistic regression analyses, the dependent variables were “ever heard about nylon teeth”, “believing in nylon teeth myth”, “nylon teeth belief ever existed in the village/area”, “information on whether during the last 2 years any child in family, close relative or friend was suspected to have developed nylon teeth or to have died of nylon teeth”, “reasons for abandoning the practice”, “modern medicine perceived as best treatment of nylon teeth” and “traditional medicine perceived as best treatment of nylon teeth”. All had responses of “Yes = 1” or “No = 0”. Respondents who reported to have never heard about nylon teeth myth were not further questioned about the myth.

Region, sex, age, education, and profession were used as the independent variables and they were included into the multivariate logistic regression analyses. These variables were dichotomized as follows: *Region* “those where nylon teeth myth was known before 1990s” dummy coded 1; “those where nylon teeth was not known before 1990s” and dummy coded 0. *Sex:* male = 0; female = 1. *Age: 18–45 years = 0; 46–98 years =* 1. *Education*: ≤ primary education =1; ≥ secondary education = 0; *Profession:* hospital workers = 0; non- hospital workers =1. In logistic regression analyses, the referent categories for independent variables were coded 0; and the outcomes of interest for dependent variables were coded 1. The level of significance for Chi-square and logistic regression analysis was set at *p*-value of <0.05. Data was entered in a computer and analyzed using SPSS version 16.

## Results

From our sampling frame, at some study sites 25 participants were not obtained from traditional healers, teachers and health workers strata. A total of 1359 respondents aged 17 to 80 years from six regions representing the six geographical zones of Tanzania participated in the study. About 58% of them were females, two thirds (66.8%) belonged to child bearing age, 61.5% had primary education or lower and 84.8% were non-hospital workers (Table 1).

**Table 1:** Distribution of 1359 respondents by demographic characteristics

Demographic characteristics	n	Percent
Region		
Nylon teeth not known before 1990s	754	55.5
Nylon teeth known before 1990s	605	44.5
Sex		
Male	576	42.4
Female	783	57.6
Age-groups		
17–45 years (child bearing age)	908	66.8
46–98 years (elders)	451	33.2
Education		
Primary education and below	836	61.5
Secondary education and above	523	38.5
Profession		
Non-hospital workers	1152	84.8
Hospital workers	207	15.2

Table 2 presents distribution of participants by responses to specific questions related to nylon teeth myth. Forty five percent (*n* = 614) reported to have heard of nylon teeth myth, of whom 283 (46.1%) believed that nylon teeth is a reality and not just a belief, and 262 (42.7%) reported that the myth was in existence at the time of study. Of those who reported that the myth still exists in their area; 51.5% and 11.5% respectively reported to have heard a child to have developed nylon teeth or died due to the myth in their family or close friends during the past 2 years.

**Table 2:** Distribution of respondents by specific questions related to nylon teeth myth

History of nylon teeth	Number	Percent
Have you ever heard about nylon teeth?		
Yes	614	45.2
No	745	54.8
	1359	100.0
If yes, are nylon teeth a reality or just a belief?		
Reality	283	46.1
Just a belief	240	39.1
I don’t know	91	14.8
Total	614	100.0
Has the nylon teeth belief ever existed in this village/area?		
Never existed	165	26.9
Existed but disappeared	187	30.5
Exists now	262	42.7
Total	614	100.0
During the last 2 years, have any child in your family, close relative or friend suspected to have developed nylon teeth?		
Yes	135	51.5
No	127	48.5
Total	262	100.0
During the last 2 years, have any child in your family, close relative or friend believed to have died of nylon teeth?		
Yes	30	11.5
No	232	88.5
Total	262	100.0

A bit more than 30 % (*n* = 187) of the participants reported that the myth was once existing in their locale but has disappeared. The reported reasons for disappearance of the myth were; it was a fashion that became outdated (51.3%; sum of strongly agree and agree), education given by oral health professionals (60.4%) and condemnation of the myth by religious leaders (23%), (Table 3).

**Table 3:** Distribution of 187 respondents who reported that the myth was there but was abandoned by the reasons for abandoning

If the practice was there but have been abandoned, what were the reasons for abandoning the practice	Number	Percent
Religious leaders condemned the practice		
Strong agree	19	10.2
Agree	24	12.8
Neutral	60	32.1
Disagree	55	29.4
Strong disagree	29	15.5
Total	187	100.0
It happened as a fashion and became outdated		
Strong agree	38	20.3
Agree	58	31.0
Neutral	54	28.9
Disagree	25	13.4
Strong disagree	12	6.4
Total	187	100.0
Because of the health education that was given by oral health professionals		
Strong agree	60	32.1
Agree	53	28.3
Neutral	46	24.6
Disagree	15	8.0
Strong disagree	13	7.0
Total	187	100.0

About 40 % (39.7%) and 62.3% of the respondents who believed in the myth respectively reported modern medicine and traditional medicines to be the best treatment for the symptoms related to the myth (Table 4).

**Table 4:** Distribution of 262 respondents who believed that nylon teeth were a reality by their perceptions on the best treatment of nylon teeth

Nylon teeth can best be treated by	Number	Percent
Modern medicine		
Strong agree	55	21.0
Agree	49	18.7
Neutral	33	12.6
Disagree	70	26.7
Strong disagree	55	21.0
Total	262	100.0
Traditional medicine		
Strong agree	95	36.3
Agree	68	26.0
Neutral	28	10.7
Disagree	27	10.3
Strong disagree	44	16.8
Total	262	100.0

The respondents’ demographic characteristics in relation to whether they have heard about nylon teeth are presented in Table 5. Residents in the regions where nylon teeth myth was known before 1990, females, with secondary education or higher and hospital workers were more likely to report that they have heard of nylon teeth myth (χ^2^ = 32.2; *p* < 0.001; χ^2^ = 7.75; *p* < 0.01; χ^2^ = 50.933; *p* < 0.001; χ^2^ = 56.327; p < 0.001 respectively). In multiple logistic regression (Table 6) only being residents in regions where nylon teeth myth was known before 1990 (OR = 8.39 (6.50–10.83), *p* < 0.001) and/or hospital worker (OR = 2.97 (1.99–4.42), *p* < 0.001) were associated with having have heard of nylon teeth myth. On the other hand, residents in the regions where nylon teeth myth was known before 1990, with primary education or lower and non-hospital workers were more likely to report that nylon teeth myth is a reality (χ^2^ = 7.756; *p* < 0.01; χ^2^ = 25.656; *p* < 0.001; χ^2^ = 17.613; *p* < 0.001 respectively). In multiple logistic regression (Table 6) all these remained statistically significant.

**Table 5:** Distribution of respondents by demographic characteristics and whether they have ever heard about nylon teeth and believe in it

	Heard nylon teeth (*n* = 1359)		Nylon teeth is a reality (n = 614)	
Demographic characteristics	Yes	No	Yes	No
Region				
Nylon teeth not known before 1990s	177 (23.5)	577 (76.5)	66 (37.3)	111 (62.7)
Nylon teeth known before 1990s	437 (72.2)	168 (27.8)	217 (49.7)	220 (50.3)
	χ^2^ = 322	0.001	χ^2^ = 7.756	*p* = 0.005
Sex of respondents				
Male	235 (40.8)	341 (59.2)	113 (48.1)	122 (51.9)
Female	379 (48.4)	404 (51.6)	170 (44.9)	209 (55.1)
	χ^2^ = 7.75	*p* = 0.005	χ^2^ = 0.609	*p* = 0. 354
Age of respondents				
17-45 years (child bearing age)	403 (44.4)	505 (55.6)	180 (44.7)	223 (55.3)
46–98 years(elders)	211 (46.8)	240 (53.2)	103 (48.8)	108 (51.2)
	χ^2^ = 0.704	*p* = 0.402	χ^2^ = 0.986	*p* = 0.327
Education of respondents				
≤ Primary education	314 (37.6)	522 (62.4)	176 (56.1)	138 (43.9)
≥ Secondary education	300 (57.4)	223 (42.6)	107 (35.7)	193 (64.3)
	χ^2^ = 50.933	*p* = 0.001	χ^2^ = 25.656	*p* = 0.001
Profession				
Non- hospital workers	471 (40.9)	681 (59.1)	239 (50.7)	232 (49.3)
Hospital workers	143 (69.1)	64 (30.9)	44 (30.8)	99 (69.2)
	χ^2^ = 56.327	*p* = 0.001	χ^2^ = 17.613	*p* = 0.001

**Table 6:** Results of multivariate logistic regression analyses - OR (95% CI) for ever heard about nylon teeth and is nylon teeth a reality and background variables studied

Background variable studied	OR (95% CI) for Heard nylon teeth (*n* = 1359)	*p*-value	OR (95% CI) for Nylon teeth a reality (*n* = 614)	*p*-value
Regions where nylon teeth were				
Not known by 1990s	1		1	
Known by 1990s	8.39 (6.50–10.83)	0.001	1.54 (1.07–2.23)	0.021
Sex				
Male	1			
Female	1.27 (0.99–1.64)	0.100	0.91 (0.65–1.27)	0.561
Age				
17–45 yrs. (Child bearing age)	1			
46–98 yrs. (Elders)	1.102 (0.88–1.38)	0.40	1.04 (0.73–1.48)	0.819
Education				
≤ Primary education	1		1	
≥ Secondary education	1.35 (1.01–1.8)	0.042	0.52 (0.36–0.75)	0.001
Profession				
Non-hospital workers	1		1	
Hospital workers	2.97 (1.99–4.42)	0.001	0.64 (0.41–1.01)	0.053

Table 7 presents the distribution of 283 respondents who believed that nylon teeth are a reality by their demographic characteristics and perceived best treatment of nylon teeth. Proportionately more residents in regions where nylon teeth myth was not known before 1990 (*p* < 0.001), the educated (*p* < 0.001) and hospital workers (*p* < 0.001) believed that modern medicine was the best treatment for symptoms related to nylon teeth myth. These were also statistically significant in multivariate analyses (Table 8). On other hand, proportionately more residents in regions where nylon teeth myth was known before 1990 (*p* < 0.001), less educated (*p* < 0.001) and non-hospital workers (*p* < 0.001) believed that traditional medicine was the best treatment for symptoms related to nylon teeth myth. In multivariate analyses, they all remained statistically significant.

**Table 7:** Distribution of 262 respondents who believed that nylon teeth is a reality by demographic characteristics and their perceived best treatment of nylon teeth

	Nylon teeth best treated by modern medicine		Nylon teeth best treated by traditional medicine	
Demographic characteristics	Disagree	Agree	Disagree	Agree
Region				
Nylon teeth not known before 1990s	15 (31.9)	32 (68.1)	35 (74.5)	12 (25.5)
Nylon teeth known before 1990s	143 (66.5)	72 (33.5)	64 (29.8)	151 (70.2)
	χ^2^ = 19.285; *p =* 0.001		χ^2^ = 32.783	*p =* 0.001
Sex of respondents				
Male	56 (54.4)	47 (45.6)	32 (31.1)	71 (68.9)
Female	102 (64.2)	57 (35.8)	67 (42.1)	92 (57.9)
	χ^2^ = 2.499; *p =* 0.114		χ^2^ = 3.259; *p* = 0.071	
Age groups				
17–45 years (child bearing age)	114 (59.7)	77 (40.3)	78 (40.8)	113 (59.2)
46–98 years (elders)	44 (62.0)	27 (38.0)	21 (29.6)	50 (70.4)
	χ^2^ = 0.113; *p* = 0.737		χ^2^ = 2.792; *p* = 0.095	
Education				
≤ Primary education	108 (70.6)	45 (29.4)	38 (23.5)	117 (76.5)
≥ Secondary education	50 (45.9)	59 (54.1)	63 (57.8)	46 (42.2)
	χ^2^ = 16.245; *p* = 0.001		χ^2^ = 31.792; *p* = 0.001	
Profession				
Non-hospital workers	141 (67.1)	69 (32.9)	56 (26.7)	154 (62.3)
Hospital workers	17 (32.7)	35 (67.3)	43 (82.7)	9 (17.3)
	χ^2^ = 20.665; *p* = 0.001		χ^2^ = 55.651; *p* = 0.001

**Table 8:** OR (95% CI) for nylon teeth best treated by modern and traditional medicine and background variables studied (*n* = 262)

	OR (95% CI) for	*p*-value	OR (95% CI) for	*p*-value
Background variables studied	Modern medicine		Traditional medicine	
Regions where nylon teeth was				
Not known by 1990s	1		1	
Known by 1990s	0.335 (0.160–0.699)	0.004	3.793(1.693–8.499)	0.001
Sex of respondents				
Male	1		1	
Female	0.59 (0.345–1.035)	0.066	0.559 (0.298–1.046)	0.069
Age groups				
17–45 years (child bearing age)	1		1	
46–98 years (elders)	0.846 (0.461–1.553)	0.590	1.024 (0.520–2.019)	0.945
Education				
≥ Secondary education	1			
≤ Primary education	1.896(1.045–3.441)	0.035	0.492 (0.261–0.926)	0.028
Profession				
Non-hospital workers	1			
Hospital workers	2.258 (1.056–4.826)	0.035	0.142 (0.059–0.342)	0.001

## Discussion

The study participants were drawn from all zones both in urban and rural including a multicultural city to capture all possible variations related to beliefs or myths. The participants’ sex (F:M = 1.34:1) and age (Child bearing age: Elderly = 3.19:1) distributions were comparable to that of the Tanzania mainland distributions (F:M = 1.05:1, Child bearing age: Elderly = 2.01:1) respectively, according to the 2012 national census [23]. Thus, the results can be considered to represent the views of Tanzanian adults about the nylon teeth myth. However, the sampling procedure could not capture 25 participants in some strata namely; traditional healers, teachers and health care workers at some study sites which may have influenced the findings.

The current findings indicate that the nylon teeth myth is still widespread in Tanzania since 42.7% of the participants who were aware of the myth reported its current existence. The myth has also been recently reported among Kenyans by Mutai et al. [5], among Ugandans by Tirwomwe et al. [8], and among Somalians by Noman et al. [24]. Furthermore, researchers have reported the negative dental consequences of practices related to the myth among African immigrants in Israel [25], Sweden [26], UK [24], New Zealand [27] and USA [28]. Residents in the regions where nylon teeth myth was known before 1990, females, the educated and hospital workers were more likely to have heard of nylon teeth myth. For those who were resident in regions where nylon teeth myth was known before 1990, the findings may largely be explained by the fact that the myth was at its peak in the 1990s [9–12] which made everyone at that time aware of the myth and its related practices. On the other hand, the nature of hospital workers’ daily responsibilities and being educated may have influence on awareness about various events in their communities. Furthermore, females’ nursing responsibilities make them aware of children’s affairs than do males. Existence of such a myth may lead to delays in seeking medical consultation or missing correct treatment in the event of diseases associated with the myth to a sizable number of children born to parents who believe in the myth. This deprives the children their basic right of being correctly treated.

Moreover, residents in the regions where nylon teeth myth was known before 1990, the less educated and non-hospital workers were more likely to consider nylon teeth a reality. A possible explanation to this observation lies on the fact that during the 1990s, the practices associated with the myth was at its pick; therefore, residents in such regions are likely to have witnessed the practice thus likely to believe it. Similarly, the less educated and non-hospital workers are easily swayed to events related to health especially when they do not receive satisfactory explanations.

About one third of the participants reported that the myth has disappeared. Most of those reporting disappearances of the myth cited education given by oral health professionals, followed by those reporting the disappearance to be related to fashion and lastly religious leaders’ condemnation of the practice. Probably the oral health professionals played a bigger role in influencing abandonment of the myth as they were responding to community and government plea to intervene the “mystery”. Our results indicate that multi-sectoral approach against this myth is likely to succeed in eradicating it.

A sizable proportion of the respondents who believed in the myth considered traditional medicines the best treatment for the symptoms related to the myth. Residents from regions where nylon teeth myth was not known before 1990, the less educated and non-hospital workers were more likely to believe that traditional medicine was the best treatment for symptoms related to nylon teeth myth. This may point to the weakness of the current management of infectious diseases that is heavily dependent on medical model of treating the biological cause and largely ignoring the life cycle and transmission of infectious agent. It is anticipated that if the management of infectious diseases in Tanzania emphasized the control of transmission of infectious agents, the myth would have disappeared. A similar observation was made by Kikwilu & Hiza [12].This is in agreement with Mogensen [29] who stated that the removal of “false teeth” among Jop’Adhola in Uganda was never a reaction to single episodes of acute diarrhoea but rather to recurring episodes. Mogensen analyzed the social course of false teeth removal (germectomy). On the other hand, modern medicine was considered the best treatment for symptoms related to nylon teeth myth by residents in regions where nylon teeth myth was not known before 1990, the educated and hospital workers. One of the possible explanation is that communities from regions where the myth was known during its peak (1980s–1990s) are likely to have witnessed or seen children who were treated by traditional healers, therefore likely to believe in traditional medicine. The other explanation could be that the less educated and non-hospital workers use traditional medicines for other ailments and the nylon myth related conditions are not exceptions. Whereas, hospital workers are informed on the causes and treatment of diseases using modern medicines thus unlikely to believe in traditional medicine to be the best cure of the diseases associated with the myth.

## Conclusion

From the results of this study, it is concluded that the nylon teeth myth still exists in Tanzania, a substantial proportionof respondents strongly believe in the myth and consider traditional medicine the best treatment of the myth related conditions.

### Recommendations

Health education to the community utilizing a multi-sectoral approach aiming at discouraging the nylon teeth myth and its related practices is recommended.

## Additional file

Additional file 1: Nylon teeth myth and associated practices questionnaire *(NB: part of the information collected through this questionnaire was published in Tanzania Journal of Health Research Doi:* https://doi.org/10.4314/thrb.v17i2.7 *Volume 17, Number 2, April 2015 1).* (DOC 36 kb)
