# Supplementary material for: Current status of nylon teeth myth in Tanzania: a cross sectional study
Source: BMC Oral Health. 2018 Jan 10;18:9. doi: 10.1186/s12903-017-0462-6 (PMC5763609; doi:10.1186/s12903-017-0462-6)
Supplement: Additional file 1: — Nylon teeth myth and associated practices questionnaire (NB: part of the information collected through this questionnaire was published in Tanzania Journal of Health Research Doi: https://doi.org/10.4314/thrb.v17i2.7 Volume 17, Number 2, April 2015 1). (DOC 36 kb) [file 12903_2017_462_MOESM1_ESM.doc]

**Nylon teeth myth and associated practices questionnaire**

***Demographic Characteristics***

1. *Reg Number* …,
2. *Region:* (1) DSM, (2) Dodoma, (3) Kagera, (4) Manyara (5) Mtwara, (6) Rukwa,
3. *Area/Village*……………., 4. *Age (yrs)* […] 5. *Sex:* (1) Female, (2) Male,
4. *Formal education:* (1) No formal education or not completed primary education, (2) primary education, (3) ordinary level secondary education (4) advanced level secondary education, (5) diploma education, (6) university education
5. *Profession:* (1) dental (2) medical practitioners, (3) nurses; (4) pharmacists and laboratory technicians (5) teachers, (6) peasants/bussines men/women (7) traditional healers

***Nylon teeth myth/beliefs***

1. Have you ever heard about nylon teeth? (a) Yes, (b) No
2. If yes, is nylon teeth a belief or reality
   1. It is a reality
   2. It is a belief
   3. I don’t know
3. Has nylon teeth belief ever existed in this village (1) currently exists (2) existed sometimes ago, not now (3) it has never existed

***If existed, but not now, go to q26***

***If exists now,***

1. During the last ***2 years***, have any child in your family, close relative or friend suspected to have developed nylon teeth? 1) Yes, 2) No. If yes,
2. During the last ***2 years***, has any child in this village believed to have died of nylon teeth related problems? 1) Yes, 2) No.
3. In this village/area, nylon teeth problem is associated with prolonged diarrhea (a) *agree completely, (b) agree, (c) neither/nor, (d) disagree, (e) disagree completely*
4. In this village, nylon teeth problem is associated with prolonged fevers (a) *agree completely, (b) agree, (c) neither/nor, (d) disagree, (e) disagree completely*
5. In this village, nylon teeth problem is associated with prolonged cough (a) *agree completely, (b) agree, (c) neither/nor, (d) disagree, (e) disagree completely*
6. In this village, nylon teeth problem is associated with delayed growth (a) *agree completely, (b) agree, (c) neither/nor, (d) disagree, (e) disagree completely*
7. In this village, nylon teeth problem is associated with drooling (a) *agree completely, (b) agree, (c) neither/nor, (d) disagree, (e) disagree completely*
8. In this village, nylon teeth problem is associated with itching (a) *agree completely, (b) agree, (c) neither/nor, (d) disagree, (e) disagree completely*
9. In this village, nylon teeth problem is associated with excessive crying (a) *agree completely, (b) agree, (c) neither/nor, (d) disagree, (e) disagree completely*
10. In this village, nylon teeth problem is associated with difficult in sucking (a) *agree completely, (b) agree, (c) neither/nor, (d) disagree, (e) disagree completely*
11. In this village, nylon teeth problem is associated with tongue tie (a) *agree completely, (b) agree, (c) neither/nor, (d) disagree, (e) disagree completely*
12. Is there any other health problem related to nylon teeth belief? ……..
13. Does the presence of nylon teeth lead to these health problems or these health problems lead to occurrence of nylon teeth? (a) nylon teeth lead to health problems (b) health problems lead to nylon teeth (c) occur concurrently

***If nylon teeth are a reality,***

1. A child who is believed to have nylon teeth can be treated of this nylon teeth problem effectively by modern medicine at hospital. (a) agree completely (b) agree (c) neither/nor, (d) disagree, (e) disagree completely
2. A child who is believed to have nylon teeth can be treated of this nylon teeth problem effectively by traditional means. (a) agree completely (b) agree (c) neither/nor, (d) disagree, (e) disagree completely

***If nylon teeth myth was abandoned***

1. In this village/area nylon teeth myth was abandoned because of religious intervention (a) *agree completely, (b) agree, (c) neither/nor, (d) disagree, (e) disagree completely*
2. In this village/area nylon teeth myth was abandoned because of nylon teeth myth was a fashion that faded away (a) *agree completely, (b) agree, (c) neither/nor, (d) disagree, (e) disagree completely*
3. In this village/area nylon teeth myth was abandoned because health education was given by health professionals (a) *agree completely, (b) agree, (c) neither/nor, (d) disagree, (e) disagree completely*
4. Is there any other reason for abandoning the nylon teeth myth? (1) Yes, (2) No. If yes, mention

***Oral health professional’s stand***

***“Nylon teeth is just a myth, we therefore strongly advice you to ignore the myth and its associated practices. When in doubt, please consult the dental practitioners at the nearest dental clinic”.***

***Thanking you for your participation in this survey!***
